# Biotin Binding Hardly Affects Electron Transport Efficiency across Streptavidin Solid-State Junctions

**DOI:** 10.1021/acs.langmuir.2c02378

**Published:** 2023-01-17

**Authors:** Sudipta Bera, Sharada Govinda, Jerry A. Fereiro, Israel Pecht, Mordechai Sheves, David Cahen

**Affiliations:** † Department of Molecular Chemistry and Materials Science, Weizmann Institute of Science, Rehovot 7610001, Israel; ‡ The School of Chemistry, Indian Institute of Science Education and Research, Thiruvananthapuram, Maruthamala, Kerala 695551, India; § Department of Immunology and Regenerative Biology, Weizmann Institute of Science, Rehovot 7610001, Israel

## Abstract

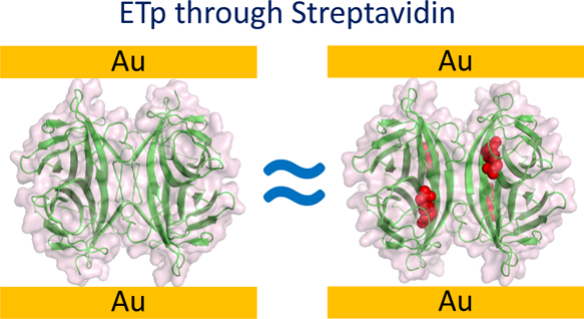

The electron transport (ETp) efficiency of solid-state protein-mediated junctions is highly influenced by the presence of electron-rich organic cofactors or transition metal ions. Hence, we chose to investigate an interesting cofactor-free non-redox protein, streptavidin (STV), which has unmatched strong binding affinity for an organic small-molecule ligand, biotin, which lacks any electron-rich features. We describe for the first time meso-scale ETp via electrical junctions of STV monolayers and focus on the question of whether the rate of ETp across both native and thiolated STV monolayers is influenced by ligand binding, a process that we show to cause some structural conformation changes in the STV monolayers. Au nanowire-electrode–protein monolayer–microelectrode junctions, fabricated by modifying an earlier procedure to improve the yields of usable junctions, were employed for ETp measurements. Our results on compactly integrated, dense, uniform, ∼3 nm thick STV monolayers indicate that, notwithstanding the slight structural changes in the STV monolayers upon biotin binding, there is no statistically significant conductance change between the free STV and that bound to biotin. The ETp temperature (T) dependence over the 80–300 K range is very small but with an unusual, slightly negative (metallic-like) dependence toward room temperature. Such dependence can be accounted for by the reversible structural shrinkage of the STV at temperatures below 160 K.

## 1 Introduction

Understanding the mechanism of electron transport (ETp) via protein molecules employed in a solid-state electrode–molecule–electrode configuration is of interest for two main reasons. One is the fundamental question, raised by earlier studies showing that ultrathin films of protein monolayers have shown electronic conductance comparable to that of conjugated organic molecules,^1^ is how efficiently can proteins conduct electronic current? The second one concerns the potential use of proteins as active components in electronic devices,^2^ which is a fascinating possibility, given their diverse properties and functions.^3^

ETp has already been studied via several different types of proteins, both redox-active ones known for their biological electron transfer (ET) function, like Azurin,^4,5^ cytochrome C (CytC),^6^ and photosystem I,^7,8^ as well as redox-inactive ones like the ion pumping bacterio-^9^ and halo-rhodopsins,^10^ the metal-oxide core-containing ferritin^11,12^ and its homologues with different metals,^13^ and human or bovine serum albumins (HSA^14^ and BSA^15^).

Issues that were found to affect the ETp across protein monolayers include the mode of protein binding to the substrate electrode, protein orientation with respect to the substrate,^3,6,7,16^ and the strength of the electronic coupling of the protein to the electrodes. This last parameter was shown to be modulated by the insertion of different (organic) linker molecules between electrodes and proteins.^5,6,17^ Furthermore, different types of protein-bound cofactors were found to enhance the ETp, making it a more efficient medium than its apo-form. Thus, a heme-depleted CytC derivative has a 3 orders of magnitude lower ETp efficiency than the holo-form at room temperature.^18^ Holo-azurin shows 2–3 orders of magnitude higher conductance than its Cu(II)-depleted form below 200 K.^4^ Furthermore, the ETp efficiency of a complex of HSA with retinoate is 2 orders of magnitude higher than that of free HSA at RT.^14^

**Figure 1:**
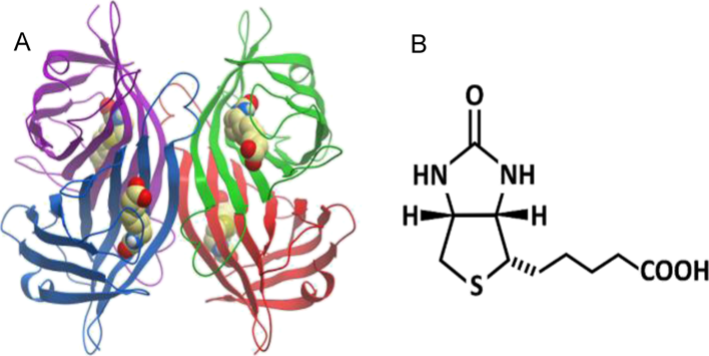
(A) Structure of biotin-bound *n-*STV protein (PDB file 6J6J) and (B) the chemical structure of biotin.

Here, we report the results of experiments aimed at examining the possible effect on the ETp via a protein upon binding its specific ligand that is not electron-rich. The ability of the protein to bind a specific ligand is a process central to the wide range of functions of proteins from enzyme catalysis to control of biochemical processes. Streptavidin (STV) is a homo-tetrameric bacterial protein known to exhibit an exceptionally high binding affinity for its ligand, the vitamin biotin (*K*_d_ ∼10^–14^ mol/L).^19^ STV’s quaternary structure consists of four identical β-barrel subunits, each composed of eight antiparallel β-sheets. Each subunit has a biotin-binding site; therefore, a single streptavidin molecule is capable of binding up to four biotin molecules (Figure 1). The exceptional affinity of STV for biotin originates from an extensive array of hydrogen bonds and van der Waals interactions between biotin and specific amino acid residues located inside a cavity that is present in each β-barrel structure of an STV subunit.^20^ In aqueous solution, before biotin binding, its site is occupied by several water molecules.^21^ During the binding of biotin, the bound water molecules are displaced, thus burying the biotin in the protein’s interior and leaving only its carboxylate group accessible to the solvent. The structural changes in the biotin-binding pockets also result in changes in the net quaternary structure of the STV-biotin complex. Thus, the biotin-streptavidin system provides an interesting model for studying if ETp via proteins is affected by ligand binding per se or only if the ligand is electron-rich.

Earlier electron transport/transfer studies were conducted on STV in an aqueous solution under non-redox conditions imposed by an electrochemical STM.^22−25^ As such, those experiments bear some similarity to ETp measurements, which also do not involve a redox process. They are, although, electrically quite different from measurements via the solid-state junction that yield ETp values because of the presence in the former of a liquid electrolyte with its ionic and polar electrical screening capabilities. These single-molecule experiments resolved statistically significant conductance differences (from 2 or 20 times for thiolated STV or thiolated biotin binding to the electrodes, respectively) between the ligand-free STV and its biotin complex.^22^ Different from those results, we now find that conductance via solid-state protein (STV-monolayer) junctions is not significantly affected by ligand (biotin) binding. Cases exist where, in a solid-state junction, a protein shows evidence of its biological conformation and some of its function,^15,26^ and, for example, electron transfer proteins appear to use some of that ability in solid-state junctions. Still, the biological activity of a protein that is immobilized on a metallic electrode will be affected, and only in rare cases we know that it is maintained (mostly in part). At the same time, the amazing chemical versatility of proteins makes solid-state junctions with them versatile. Here, we use the biotin-STV binding chemistry, maintaining as much of the relevant conditions for that binding as is known from biology (discussed in experimental). We prepared the biotin-streptavidin complex and biotin-free protein SAMs in proper biological conditions, which was then dried (except for the structural water) for experimental use. Our goal is to examine any dependence of ETp on biotin binding to STV and explore the probable mechanism of ETp across these protein junctions. We ascribe this finding to the lack of electron-rich character of biotin, which implies that the STV frontier orbital energy levels and, hence, their hybridization with the electrodes’ energy bands, are not affected significantly by ligand binding. We note that any change in the monolayer width upon biotin binding, which will, to a first approximation, affect ETp exponentially, is within our experimental errors. Our results imply that results of the STM-in-electrolyte measurements are likely due to changes in the electrical screening of the protein’s surface charges by the electrolyte caused by biotin binding. Interestingly, we observed a small negative temperature dependence in conductance behavior for both STV and its biotin-complex monolayer above ∼160 K and virtual temperature independence below 160 K.

## 2 Experimental Section

### 2.1 Reagents and Solution Preparation

Both native streptavidin (*n*-STV) and thiolated streptavidin (*t*-STV) were used in the ligand-free form as well as in the biotin-bound state. *n*-STV, *Streptomyces avidinii*, a recombinant product expressed in *E. coli*, was procured from Sigma. Native STV lacks exposed cysteine residues; therefore, a commercially available thiol-modified STV (*t*-STV) purchased from Protein Mods has been used. This thiolated STV (*t*-STV) protein molecule is reported to have an average of ∼2.5 surface-exposed thiol groups prepared by chemical coupling of 2-iminothiolane and the solvent-accessible amine groups of *n*-STV. To prevent inter-protein S–S linking, only few imine carbons (of 2-iminothiolane) were attached to free amine (-NH_2_) groups of the Lys residues. There are 16 Lys residues on STV, which are all surface-exposed and distributed over the whole protein (PDB–6J6K). Therefore, the introduced thiols are positioned randomly over the STV surface.

Solutions of 1 μM *n*-STV were prepared in phosphate buffer pH 7.1 (1 mM phosphate) and similarly for *t*-STV in pH 8.0 (10 mM phosphate and 150 mM NaCl). These were used for preparing self-assembled monolayers on gold (Au), a 50 nm film of which was evaporated on Si as a substrate(Au/Si). The biotin STV complexes were prepared by mixing protein and biotin (in μM concentrations) at a 1:4 molar-ratio for *n*-STV and 1:5 for *t*-STV (in the respective phosphate buffers); the mixture was kept for 30 min under gentle stirring at 4 °C. Freshly prepared biotin-STV complexes were used for monolayer preparation and were characterized as detailed below.

### 2.2 Protein Monolayer Preparation

Both electrostatic and covalent binding of the protein to the substrate electrode were used for monolayer preparation. First, protein monolayer formation was optimized on the Au/Si substrate; the optimized procedure was then used to deposit protein monolayers on microfabricated Au electrodes.^16,27^ The Au/Si substrate was first cleaned by bath-sonication, sequentially in acetone, isopropyl alcohol, and double-distilled water for 10 min each, followed by drying in a dry N_2_ stream. Next, the N_2_-dried substrate was cleaned by UV-generated O_3_ for 10 min. For electrostatic immobilization of the *n*-STV or its biotin complex, the ozone-cleaned Au substrate was cleaned in hot ethanol for 20 min and followed by N_2_ drying. It was then immediately dropped into ethanolic solution of cysteamine (∼2 mM) and left overnight (15–17 h). The cysteamine-modified Au substrate was sonicated in ethanol for 3 min to remove loosely bound cysteamine from the top of the linker layer and then washed thoroughly with excess water. Then, after N_2_ drying, the cysteamine-coated Au substrate was incubated in 1 μM *n*-STV (or *n*-STV-biotin complex) solution in pH 7.1 phosphate buffer for about 1 h in a humidity chamber at room temperature. After water washing and N_2_ drying, a monolayer of *n*-STV (or its biotin complex) on Au was obtained.

For preparing monolayers of the thiolated STV, the solvent-cleaned, ozone-activated Au substrate was immersed directly in a 1 μM *t*-STV in phosphate buffer pH 8.0 solution for 2 h. After water-washing and N_2_-drying, the protein monolayer was ready for characterization and measurements. A similar immobilization procedure was followed for preparing the biotin-*t*-STV complex monolayer.

### 2.3 Monolayer Characterization

Protein layer uniformity and surface coverage were tested by tapping mode AFM imaging and surface topography analysis. Protein monolayer thickness was determined by ellipsometry and AFM nano-shaving (see SI). The structural properties of the protein monolayers were further studied by polarization modulation-infrared reflection-adsorption spectroscopy, (PM-IRRAS) (see SI). Work function values (ϕ) to assess the surface charge were obtained from Kelvin probe-based contact potential difference (CPD) measurements; these measurements also served to compare between monolayers of streptavidin and its complex with biotin. The work functions of the protein monolayer-covered substrates were determined relative to the known work function of freshly cleaved HOPG (highly ordered pyrolytic graphite) (4.6 eV)^28^ (see SI).

### 2.4 Nanowire Trapping for Junction Fabrication

**Figure 2:**
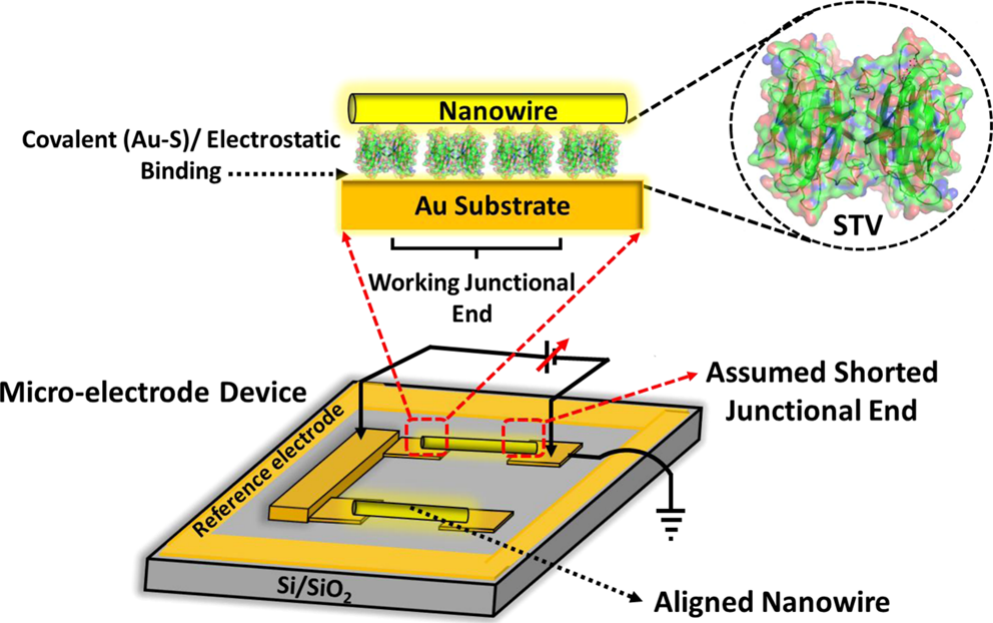
Cartoon representation of the Au – *streptavidin (STV)* – AuNW (in yellow) junction configuration for the probe station-based *I*–*V* measurements. Further explanations of the device structure are in the main text.

The fabrication procedure of the suspended gold-nanowire (AuNW)-based, non-destructive, top-electrode contact has been described in detail elsewhere.^5,27^ Here, we slightly modified the procedure to increase the fraction of junctions where only a single AuNW is trapped within an array of microfabricated electrode devices (based on a chip developed by Noy et al.^29^) from ∼10 to ∼35% of all possible junctions on the chip. First, monolayers of the *n*-STV and *t*-STV proteins (or their respective biotin complexes) were prepared on the chip’s microelectrodes (bottom-electrode) as described above. AC field-induced dielectrophoretic trapping of the AuNWs was used to deposit the AuNW as a top-electrode contact on the protein-modified chip. This yielded permanent Au–protein–Au junctions (Figures 2 and S1), which were used for the ETp study. We modified the process compared to our previous studies^27^ by using a larger drop volume (70–90 μL) and a (10×) more diluted aqueous suspension of AuNWs for the trapping process. In addition, a relatively high AC bias (V_p-p_ = 4.5 V) was applied (by an AC function generator model 8122, Tabor Electronics Ltd.) to generate a non-homogeneous AC field across the devices. We then continued with the established procedure^5^ and after NW trapping, the chip was rinsed with a gentle water flow and dried under a low N_2_ flow. With this modification, we obtained 90–120 (out of 260) devices on a single micro-electrode chip, instead of 20–30 with the original procedure. Among these, not all had a single-AuNW trapped since some had multiple AuNW bridging-contacts and were not subjected to ETp measurement. About 60–80 single AuNW junctions were available for ETp measurements. The specific coordinates on the chip’s grid of all junctions with a single-trapped AuNW were determined by optical microscopy at 50X magnification.

### 2.5 Current–Voltage Response Measurements

To carry out current–voltage measurements across the Au-protein-AuNW junctions, the lithographically fabricated micro-electrode devices^27,29^ were loaded inside a cryogenic probe station (Lakeshore TTPX) under 10^–5^ mbar vacuum pressure and kept there overnight. Current response measurements were carried out across the protein monolayer (highlighted in Figure 2) using a sub-Femtoamp source meter (Keithley 6430). I–V measurements were done for every single-nanowire junction at room temperature (293 ± 2 K) under ±0.5 V bias. As shown earlier, one end of the NW shorts to the Au pad on the chip in nearly all single NW junctions so that only a single Au-protein-AuNW (Figure 2) junction remains, likely caused by the hard landing of one end after which the second end of the NW lands softly.^30^ The I–V measurements were controlled with dedicated LabView-based programmable software. The scan was from 0 → −0.5 → +0.5 → 0 V, with a sweep rate of 20 mV/s. Based on the bias-dependent current responses, some junctions are classified as non-contact (current <10 pA @0.5 V), or shorted (current >1 mA @0.5 V), and the remaining are considered as active ones (10 pA < *I* < 50 μA @0.5 V). However, most junctions that pass >50 nA currents at 0.5 V were found to be shorted after a few scans and are considered to be partially shorted.^27^ The junctions with lower currents (30 pA < *I* < 50 nA @ 0.5 V) were noticeably less noisy than junctions with higher currents. These junctions also gave less noisy and more stable *I*–*V* traces and had insignificant *I*–*V* hysteresis. Therefore, these junctions were selected for measuring the temperature dependence of the *I*–*V* characteristics (*I*–*V*–*T*). All measurements (of *t*-STV) were started from 300 K, cooling down slowly (stepwise with liquid N_2_) to 80 K, with measurements at intermediate temperatures (280–240–200–160–120–100–80 K) at 10^–5^–10^–6^ mbar. The junction was then gradually heated up, and ETp was measured at the above intermediate temperatures in reverse order up to 300 K. Similarly, such a reversible temperature dependence study has been done on *n*-STV junctions by cycling (once) between 300, 200, and 110 K.

### 2.6 AuNW Junction-Based Current Measurements

The average AuNW dimensions are 3–5 μm long with a ∼250 nm diameter; still, due to breaking of AuNWs during bath sonication, the length of the trapped AuNWs varies within the above dimensions (diameters remain unchanged). This variation cannot explain the order of magnitude variation in the currents’ size observed for the non-shorted Au-protein-AuNW junctions (see the junctions’ current variation mentioned in ‘results and discussion’). The experimental range of currents can be partially correlated with the variation of the effective electric contact area, which, for larger area contacts (∼10^5^ μm^2^), can well be up to 6 orders of magnitude smaller than the geometric area^31^ of any electrical contact; even for the AuNWs (≥∼ 5 × 10^3^ nm^2^ geometric contact area), the actual electrical contact area was still estimated to be smaller (∼10–100×) than the geometric contact area.^32^ The finding that there is still a high current variation via AuNWs can be explained by considering both the variation of the electrical contact area and the nano-wire penetration into the protein layer. Protein layer defects and possible variations in the dielectrophoretic force distribution may result in some penetration of the AuNW (during the trapping) through the soft-protein monolayer, which can change the effective electric contact area and reduce the separation between top and bottom electrodes. One important cause is that the applied AC field between the reference and working electrodes^27^ will vary somewhat from one electrode to another due to differences in the distance between them in the macroscopic (∼5 × 5 mm^2^) chip (Figure S2). The nanowire trapping force (dielectrophoretic) is directly proportional to the square of the applied electric field.^33^ This will govern the extent of AuNW penetration, if there is any, and will, in extreme cases, lead to shorted junctions. Hence, the nanowire configuration poses challenges to obtaining a stable current with a narrower junction current distribution due to its characteristic bias-dependent current response via a protein layer.

### 2.7 Statistical Data Analysis

For each protein type, about 100 single AuNW trapped junctions (micro-electrode devices) of different proteins and their complexes (active junctions) were measured to get statistics for the currents at a specific bias (0.5 V). For each type of active protein junctions, 6–9 chips were studied in order to get reliable statistics (see in discussion). To identify the characteristic STV-monolayer junction-currents, data analysis was done for the *I*–*V* responses of randomly selected active junctions from both the same chip (with multiple micro-electrode devices) and from different chips (both from chips made in the same batch and from chips of different batches). The results yield a statistical data variability of mesoscale protein junctions in terms of specific bias-dependent current histograms.

## 3 Results and Discussion

**Figure 3:**
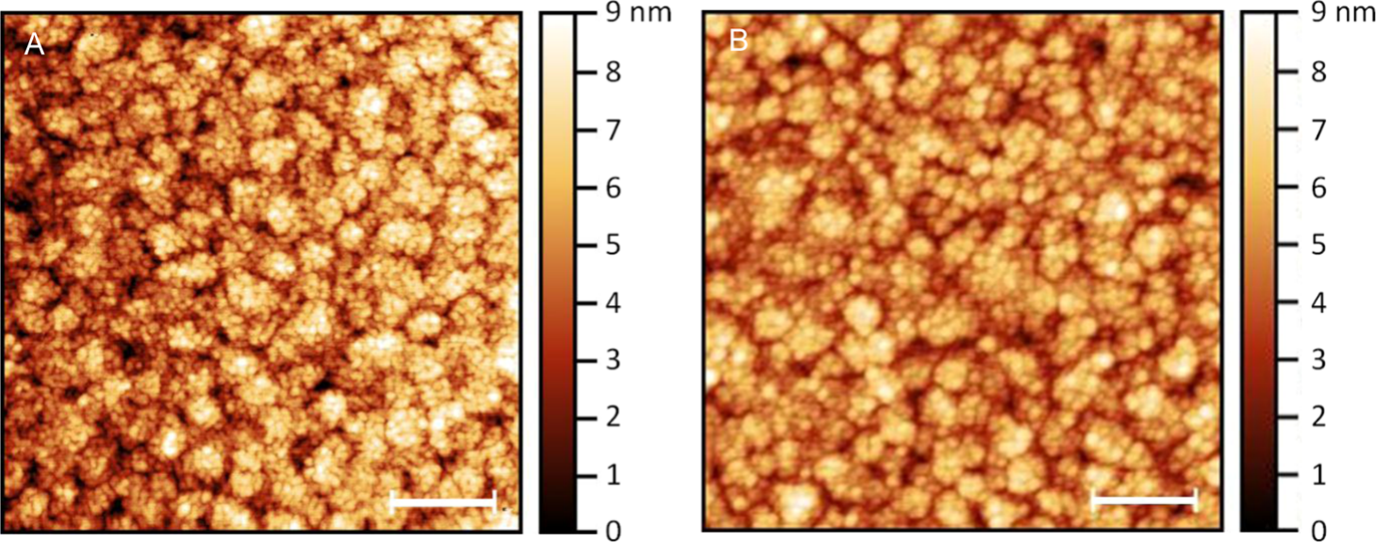
AFM topography of the protein monolayers on the Au/Si substrate, (A) ***t*-STV** (*rms* roughness 1.3 nm), and (B) ***t*-STV complex** (*rms* roughness 1.0 nm). Scale bars are 200 nm.

ETp was measured via junctions with monolayers of the two different streptavidin forms and of their respective different biotin complexes. The protein was bound to the underlying Au micro-electrode, either electrostatically for *n*-STV or covalently with thiolated streptavidin (*t*-STV). The electrostatic binding of *n*-STV was done by the interaction of the net negatively charged protein surface with the positively charged cysteamine monolayer bound to the Au electrode by Au–S bonds. The employed experimental pH (7.1) was above the isoelectric point of *n*-STV, which ensures a net protein negative surface charge. *t*-STV was covalently (Au–S) bonded to the electrode at pH 8.0.^34^ For *n*-STV, electrostatic adsorption leads to the random orientation of individual protein molecules on the surface. Similarly for *t*-STV, the randomly oriented thiol groups (explained in experimental) do not allow for an oriented configuration. More uniform and dense protein coverage of the electrodes was obtained with *t*-STV than for *n*-STV. Self-assembly of the former and its biotin complex monolayers yielded a dense, homogeneous protein film on the Au surface (Figure 3). The thickness values of the monolayers, deduced from ellipsometry, were 2.6 ± 0.2 nm for *t*-STV and 2.7 ± 0.2 nm for the *t*-STV-biotin complex (see Table 1). In contrast, it was very difficult to obtain uniform coverage of the *n*-STV monolayer (∼2 nm) on a large Au area (few mm; beam size of the ellipsometer). Still, some areas have been found with a good uniform coverage of both *n*-STV and its biotin complex (Figure S3) monolayer on gold. This was evident, especially for its biotin complex (∼1.6 nm). The poorer quality of the latter monolayers can explain the lower ellipsometric (averaged) thickness of *n*-STV monolayers rather than of the *t*-STV ones (Table 1). We note that, notwithstanding several-year-long, multi-person efforts, we were unable to prepare the biotin-STV complex monolayer by immobilizing the *n*-STV molecules on top of thiolated biotin-bound Au-substrates, suitable for electrical transport measurements. The binding process could not take place probably due to steric hindrance since the biotin-cavity of streptavidin is far away from the active side of thiolated-biotin, which is a comparatively shorter molecule.

**Table 1:** Thickness of the protein monolayers deduced from ellipsometry and AFM nano-shaving experiments

	protein monolayer thickness(nm)			
	*n*-STV		*t*-STV	
protein layer	ellipsometry	AFM nano-shaving	ellipsometry	AFM nano-shaving
STV	2.0 ± 0.2	2.2 ± 0.1	2.6 ± 0.2	3.2 ± 0.4
complex	1.6 ± 0.3	2.0 ± 0.2	2.7 ± 0.2	3.5 ± 0.2

Based on tapping mode AFM topography images made on the few μm scale, a relatively sufficient good enough surface coverage was obtained with *n*-STV and its biotin complex (Figure S3), enabling reliable I–V measurements. Also, uniform, aggregation-free, homogeneous surface topography of *t*-STV and its biotin complex monolayers was observed by tapping mode AFM imaging (Figure 3). In parallel, the thickness of the protein monolayer was estimated from image analysis after nano-shaving by the AFM tip. From the results of several monolayers, the protein monolayer thickness was 3.2 ± 0.4 nm for *t*-STV and 3.5 ± 0.5 nm for the *t*-STV-biotin complex (see Table 1; Figure S4 shows the examples of these experiments).

With *n*-STV, it was, however, difficult to obtain reliable measurements of the thickness of electrostatically bound monolayers or of the STV-biotin-complex. The reason appears to be that the *n*-STV layer behaves as a sticky material, which, in nearly all cases, cannot be removed completely from any region, on which most of the nanoshaving was attempted. Still, persistence paid off and a few good nano-shaved areas were obtained and showed ∼2 nm protein thickness (Figure S5). The calculated thickness of the *n*-STV monolayer (by AFM nano-shaving) supports the thickness values derived from ellipsometry (Table 1). We suggest that the difference between the *n*- and *t*-STV samples is due to the different nature and strengths of their interactions with the substrate, as no stickiness was observed for the covalently bound *t*-STV. Because of this problem with *n*-STV, monolayer width comparisons with *t*-STV samples are based on ellipsometry results.

As can be seen from its crystal structure (PDB–6J6K), tetrameric streptavidin is not a completely symmetrical protein (Figure S6) as it has a ∼5.4 nm long and a 4.7 nm short axis. To obtain the thickness of monolayers, AFM nano-shaving (direct approach) is considered a more reliable method than ellipsometry (being indirect, using fitting optical data to a model). Comparing the thickness determined by nano-shaving with the X-ray data shows a ∼1.5–2.5 nm smaller thickness than the lowest possible dimension (4.7 nm short axis), which rules out a tilted orientation of the bound STV. Therefore, the difference in monolayer thickness may possibly originate from the immobilization onto the Au substrate, possibly causing some tetramer dissociation into a monomer or a dimer.

**Figure 4:**
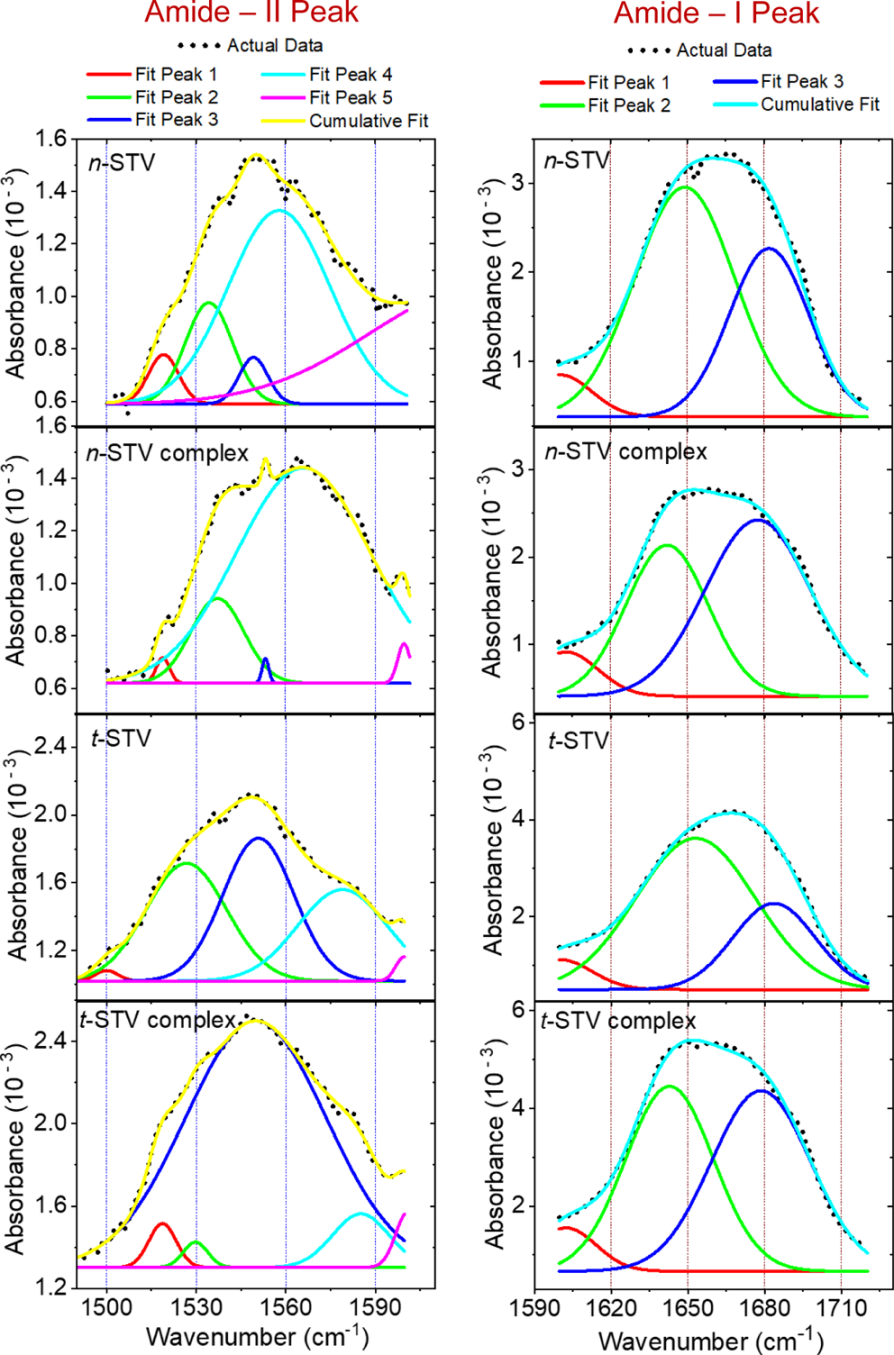
Deconvoluted amide-I (**right** column) and amide-II (**left** column) IR absorption bands of STV and its biotin complex obtained from PMIRRAS spectra of different protein monolayers mentioned in each figure. The deconvoluted amide bands consist of several peaks—five peaks for amide-II and three peaks for amide-I bands. The dotted curves are the actual data, and the solid curves are the fitted ones, according to the legend for the different colors at the top of each column.

In their PM-IRRAS spectra, characteristic amide bands of the protein monolayers was observed. Biotin-free *n*-STV and *t*-STV have similar amide-I and amide-II bands at ∼1666 and ∼1550 cm^–1^, respectively (Figure S7). Biotin binding induces a clear change in the shape of the amide-I band, where the band-maxima shift to a smaller wavenumber for both *n*-STV (∼1660 cm^–1^) and *t*-STV (∼1657 cm^–1^) (Figure S7). Following binding of biotin, the amide-II band was broadened for both *n*-STV and *t*-STV (Figures S7,and 4). For clarity, the individual bands were deconvoluted for each type of protein layer. Five different arbitrarily chosen^35^ peak positions were selected for amide-II and three for amide-I bands, as shown in Figure 4. A qualitative distinction can be made between free and biotin-bound streptavidin for both native and thiolated proteins. The ratio of the areas under the deconvoluted curves clearly varies between free and biotin-bound STVs. In the amide-I band (right column in Figure 4), the area-ratios of deconvoluted curves (with peak positions at about 1645 and 1680 cm^–1^) are affected noticeably for both types of STV.

Biotin binding causes the relative contribution of the curve (peak @1680 cm^–1^) to be significantly enhanced compared to the curve that peaks at about 1645 cm^–1^. In the case of the amide-II band, an even more prominent distinction can be seen for the effect of biotin binding. For *t*-STV, the contribution of the curve with the peak @1550 cm^–1^ reaches a maximum for the biotin complex compared to the biotin-free state. Similarly, for *n*-STV, upon biotin binding, the deconvoluted curve (with a peak of about 1560 cm^–1^) contributes more to the amide-II band than the band of the protein in its biotin-free state (left column in Figure 4). Therefore, the change in the area ratio of the deconvoluted curves clearly illustrates the effect of biotin binding to STV in a monolayer-on-substrate form, similar to an earlier solution-based study.^35^

The amide-I and amide-II bands of proteins represent a mixture of protein conformations, which control their position and shapes (Figure S7), mainly due to different hydrogen bonding networks to the carbonyl and/or N–H groups. The fact that biotin binding changes, especially the shape of the amide-I band, indicates that it induces protein conformational alterations, thereby changing the contribution of different amide bands to the final amide band. It was previously shown that binding of biotin to streptavidin in aqueous solution changes the protein conformation.^21^ Indeed, the PMIRRAS spectral results agree with the expected effect of biotin binding and show that the features observed in solution^21^ are retained in the solid-state biotin-STV complex monolayer. The different PMIRRAS spectra of the biotin complexes of *n*-STV and *t*-STV indicate that the two complexes adopt different conformations.

CPD measurements provide information about the relative surface charge densities of the protein layers. The results show a notable difference between the biotin-complexes and the free proteins of both *n*- and *t*-STV. Monolayers of both ligand-free proteins on Au have a ∼4.75 eV work function, which indicates that the 2-iminothiolane modification of the *n*-STV caused no measurable change in the net *t*-STV surface charge. In addition, the different pH values (7.1 and 8.0) employed for monolayer preparation did not affect the streptavidin work function (Table 2). In contrast, there is a clear difference in work function, was observed for the STV-biotin complex monolayers on Au : 4.70–4.72 eV with *n*-STV and 4.82–4.87 eV *t*-STV (Table 2). This may reflect slightly different protein conformations of their complexes with biotin (Figure S8), as also indicated by the PMIRRAS spectra (Figures 4,and S7). It is known that the biotin carboxylate sticks out of the β-barrel pocket of each streptavidin subunit and is stabilized by nearby (for *n*-STV) surface-exposed basic amino acid (−NH_2_ terminated) residues (blue highlighted around the biotin-binding cavity in Figure S8). However, in the case of *t*-STV, several exposed basic amino acid residues are derivatized by the thiol-terminated linker (2-iminothiolane). This may affect the overall stabilization of the surface-exposed carboxylates of bound biotin with the nearby surface-exposed amines of the basic amino acid residues of STV. Therefore, the expected interaction of biotin’s carboxylate (in *t*-STV) with surface amines can alter the structure of the complex compared to that of the *n*-STV complex.

**Table 2:** Work function values derived from contact potential differences with respect to the freshly cleaved HOPG’s ϕ_HOPG_ = 4.6 eV as measured with a Kelvin probe

*n*-STV: Δ*V*_CPD_ [*V*_CPD_(STV)–*V*_CPD_(complex)]: 20–60 mV		
type of protein layer	*n*-STV	*n*-STV complex
work function(eV)	4.74–4.76	4.70–4.72

*t*-STV: Δ*V*_CPD_ [*V*_CPD_(complex)–*V*_CPD_(STV)]: 70–90 mV		
type of protein layer	*t*-STV	*t*-STV complex
work function(eV)	4.75–4.78	4.80–4.87

Two probe *I*–*V* measurements of suspended-nanowire junctions of the two types of streptavidin and their respective biotin-complex monolayers showed current values between several dozens of pA to a few tens of μA at ±0.5 V at room temperature. Junctions with currents in the 30 pA–50 nA range at ±0.5 V were monitored, as stated above (experimental section) for average current-response calculation.

**Figure 5:**
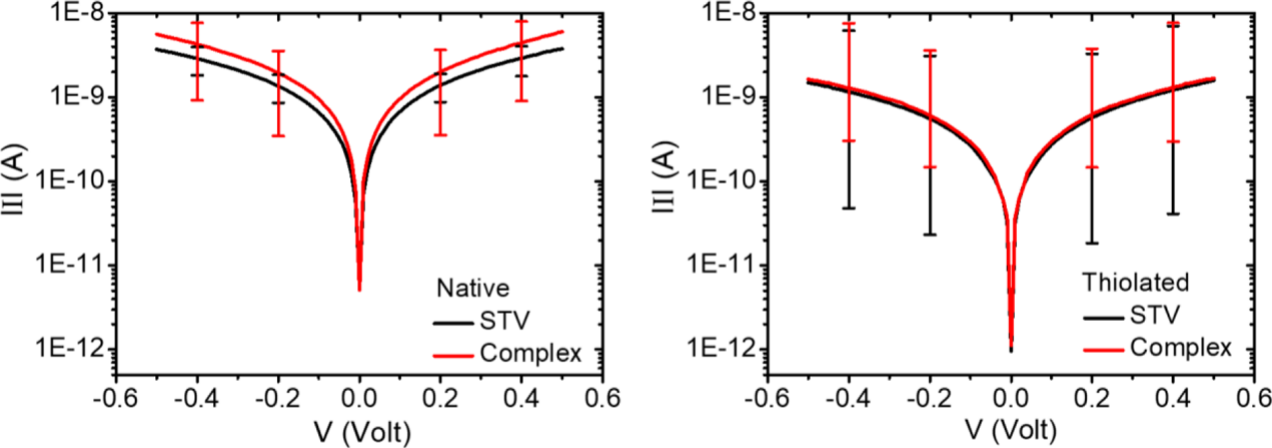
Averaged I-V responses over 40 relevant nanowire junctions (30 pA < *I* < 50 nA@0.5V) of the *n*-STV/complex (**left**) and *t*-STV/complex (**right**) with their respective error bars.

While the currents obtained for *t*-STV and *n*-STV cover a wide range, we find that the averaged values of ETp via biotin-free and biotin-bound *t*-STV and *n*-STV almost overlap (Figure 5). This holds to a lesser extent for the electrostatically bound *n*-STV compared to covalently bound *t*-STV. In the case of *n*-STV, the obtained average currents (at 0.5 V) were 3–4 times higher than with *t*-STV junctions (Figure 5). Indeed, such inter-junction current variation cannot be considered significant, as the AuNW-based protein junctions were found to exhibit a wide range of conductance. As a consequence, the error bars (conductance) overlap with one another for different sets of protein layers (Figure 5). Hence, the three- to fourfold variation in the observed current is statistically insignificant. Despite different protein monolayer widths obtained from ellipsometry (∼2.6 nm for *t*-STV and ∼2 nm for *n*-STV), real junction widths determining the separation between the two electrodes are similar ∼2.6 nm. Examining the ellipsometry thickness-based analysis for *n*-STV, the total junction width is composed of 0.5–0.6 nm for the cysteamine linker and ∼2 nm of the protein. For *t*-STV, the total layer thickness was ∼2.6 nm; but this is not only due to the protein, as thiol modification occurs via a ∼0.5 nm long thiol-terminated linker with a four-carbon spacer bound to the native *n*-STV.

**Figure 6:**
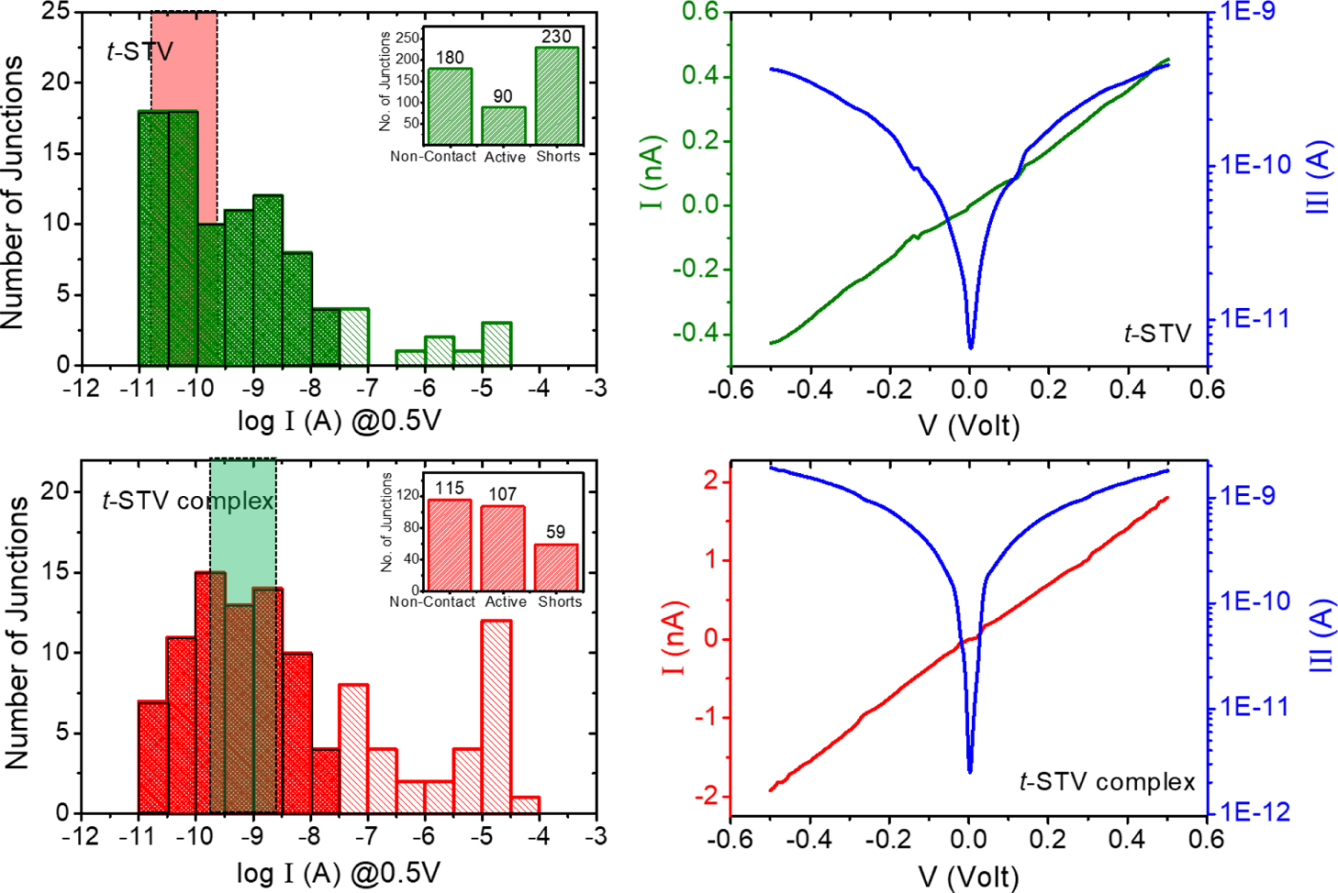
Room-temperature (293±2 K) current distribution (**left** column) of ∼ 100 Au — *protein (complex)* — AuNW junctions @ 0.5 V applied bias and the representative current–voltage responses (**right** column) of ***t*-STV (top row)** and its **biotin complex** (**bottom** row) junctions, respectively. The semi-transparent dotted-color-boxes in the left column (pink for *t*-STV and green for the *t*-STV complex) highlight the most probable junction current range for each junction type. The inserts in the left column figures give the statistics for all the junctions of each type that we measured as *active*, *non-contact* (open), or *shorted*. The part of the histogram with intense colored (green/red) bins corresponds to the *active* junctions for conductance comparisons. Junctions with > 50 nA @ 0.5 V currents are assumed to be partially shorted junctions, and open junctions < 30 pA @ 0.5 V) are excluded from the conductance comparison analysis.

The current response was symmetric with respect to the applied bias polarity. The collected values of individual junction currents are shown for ∼100 active junctions at 0.5 V at room temperature (Figure 6) of *t*-STV (and its complex) monolayers. However, the current histograms do not follow a normal distribution (Figure 6), which prevents resolving small differences between the averages obtained for different protein types. Still, the most probable junction currents (shaded parts in the Figure 6 histogram) via *t*-STV are smaller than those via the biotin-*t*-STV complex, approaching 100 pA versus few nA (@0.5 V), respectively (Figure 6). In this analysis, only junction currents up to 50 nA (@ 0.5 V) were taken into account, while those with higher values were considered as partially shorted (see in Figure 6 for the details). Unfortunately, for *n*-STV, much fewer junctions could be measured reliably due to the poor coverage on the substrate electrode surface; hence, in that case, there are insufficient data for constructing a meaningful current histogram.

Based on statistics, within our measurement precision, the differences in currents via junctions of biotin-bound and -free STV are not significant. Such is in contrast to striking differences obtained with the same type of nanowire-based junctions with proteins like Azurin (Az); the Az-linker bond switches the Etp mechanism from resonant to off-resonant tunneling,^5^ and a remarkable change in conductivity (> two orders of magnitude) was obtained in HSA after binding with retinoic acid^14^ or porphyrin^36^ in Hg/LOFO top electrode configuration.

In work that is thought to be at the single molecule level, using an electrochemical STM-based approach, where *t*-STV and its biotin-complex were bound directly to Au, as is the case here, yielded a relative conductance of the complex that was slightly less than twice that of the biotin-free protein, as derived from Gaussian distributions of data.^22^ Although no such distribution is obtained here, and while our results for *t*-STV (Figure 6) show differences between the most probable results (shaded color zones in Figure 6), those are, in our protein junction configuration, not considered significant. Biotin is a small aliphatic molecule that has no electron-rich character. Therefore, no significant change can be expected in ETp of the streptavidin junction upon binding biotin. This is because ETp is a process where protein frontier energy level alignment at the contacts with the electrodes’ Fermi level is dominant, and biotin binding should not affect those energy levels. This lack of the biotin-binding effect can be contrasted with that of the presence of an electron-rich heme in CytC,^18,37^ or of the chromophore-retinoate binding to HSA,^14^ and of Cu(II) in Azurin,^4^ where their monolayer conductance was enhanced by up to 2–3 orders of magnitude.

**Figure 7:**
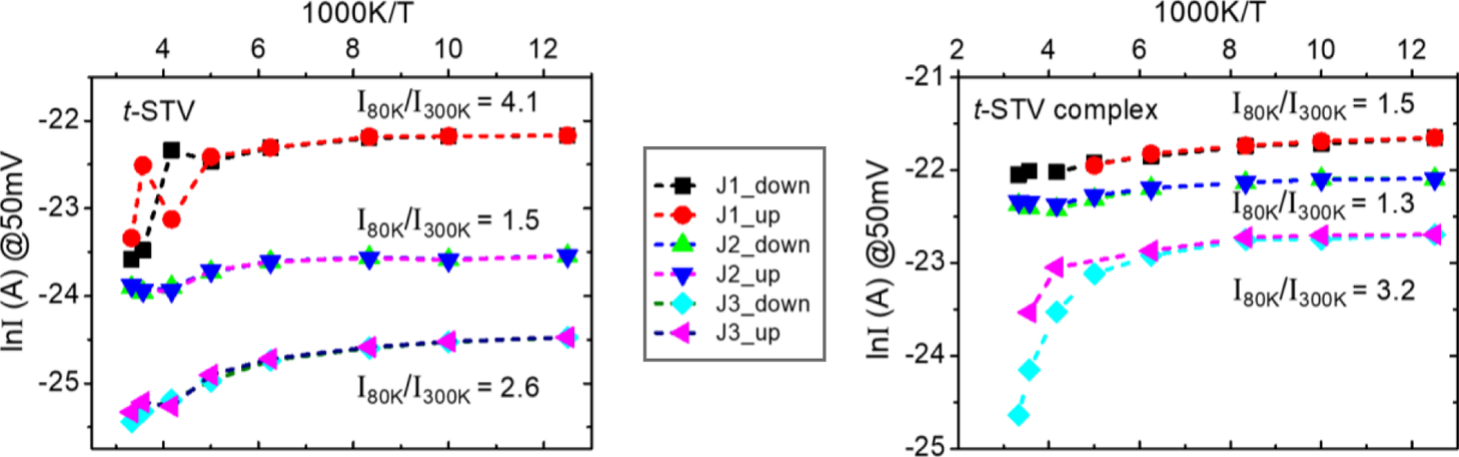
Reversible ln(I) – (1/T) characteristics of ***t-*STV** (*left*) and ***t*-STV complex** (*right*) junctions. All show qualitatively comparable temperature dependence, even if the absolute currents range over about an order of magnitude. The representative curves are shown for three different *t*-STV junctions (J1, J2, and J3) with the magnitude of current (@ 50 mV) for 300K and 80K indicated in the parentheses. For the *t*-STV junctions J1 (58-240 pA), J2 (41-60 pA), and J3(9-24 pA) and for the *t*-STV-biotin complex junctions J1 (270-400 pA), J2 (200-260 pA), and J3 (44-140 pA). In the figure, legend ‘down’ means cooling down toward low temperature, and ‘up’ means heating up to room temperature.

The observed slight difference in both the distribution and most probable junction current between *t*-STV and its biotin complex conductance (cf. Figure 6) could be due to the conformational change of the protein induced by biotin binding, as seen in the PMIRRAS and CPD (Table 2, Figures 4 and S7). Based on these results, we can assume that the *t*-STV structural changes induced by biotin binding are sufficient to yield the small conductance difference. Here, the measured Etp through STV junctions could be the combined effect of electron transport through randomly oriented protein molecules in SAM (without a preference for a particular orientation), irrespective of the immobilization type, either electrostatically or covalently (explained before). Temperature-dependent current–voltage responses were measured mainly for junctions of *t*-STV and its complex because these were more stable than those of *n*-STV (see Supporting Information, Sec 1.5). In most cases, a gradual increase in current through *t*-STV junctions was observed with decreasing temperature. This increase could, at 50 mV, reach 80 K, up to 8 times the value at room temperature (Figures 7 and S9). Such negative temperature-dependent *I*–*V* responses were observed for more than 70% of junctions (Figure S9), and all *t*-STV junctions that showed reversible temperature dependence (RT → low temperature → RT) showed this negative temperature dependence.

Negative temperature dependence (up to 2×) was also found for several of the measured *n*-STV junctions (See Supporting Information Figure S10). In reverse temperature dependence measurement, the biotin-free *n*-STV junction showed a consistent slightly negative temperature dependence. However, biotin-bound *n*-STV exhibited somewhat scattered, but close to reversible temperature, dependence over the 300 to 110 K temperature range (Figure S10).

About 30% of the *t*-STV junctions showed some significant current decrease from 300 to 80 K, that is, a positive temperature dependence. However, none of those junctions were sufficiently stable for the reversible *I*–*V*–*T* study; rather, they yielded very scattered current values with shorted or open junctions. An open junction means that, after cooling down, the junction did not respond anymore during subsequent heating back to 300 K. Thus, the small but quite reproducible negative temperature dependence over the 160–300 K temperature range (on the average 2-3× increase) (Figures 7 and S9) seems characteristic for stable *t*-STV junctions.

**Figure 8:**
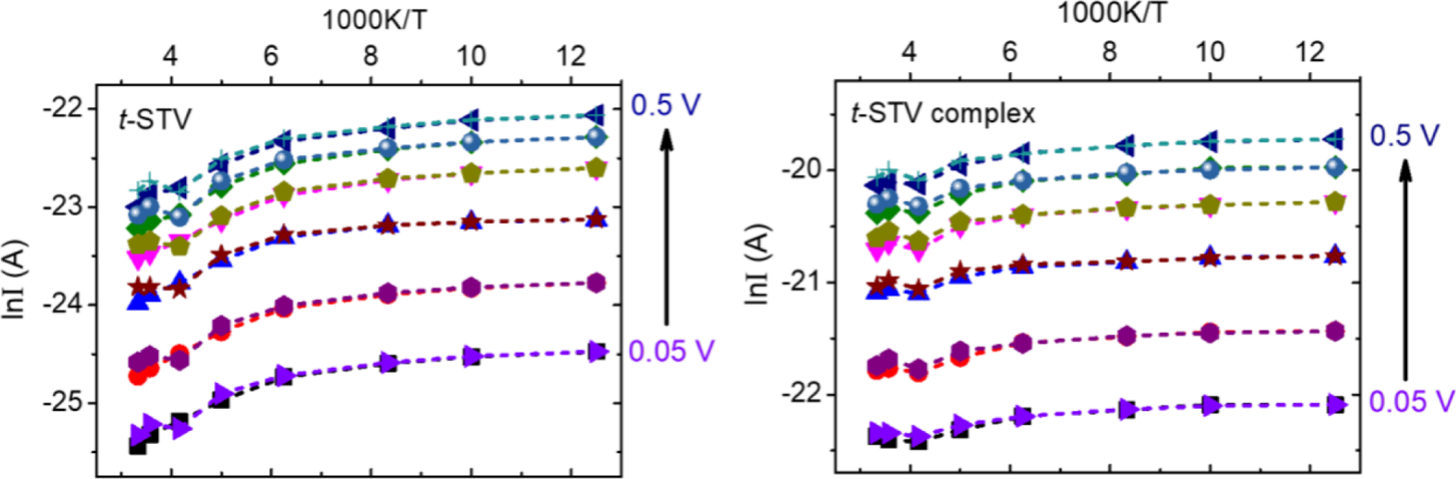
Characteristic temperature-dependent current response plotted as ln(I) – (1/T) at different applied bias voltages from 0.05 to 0.5 V (0.05, 0.1, 0.2, 0.3, 0.4, and 0.5 V) of junctions with monolayers of ***t-*STV** and its biotin **complex** in the nanowire junction configuration, showing inverse, that is, positive temperature dependence. For each bias, the temperature-dependent response consists of two dotted lines, one for cooling down to low temperature and another one for heating up to room temperature.

The reversible temperature-dependent *I*–*V* responses (*I*–*V*–*T*) shown in Figure 8 are representative of *t*-STV junctions; the corresponding *I*–*V* plots are presented in Figure S11. The temperature-dependent ETp of biotin-free *t*-STV junctions was mostly similar to that of the biotin-bound *t*-STV junctions. Junctions of biotin-free *t*-STV show slightly stronger temperature dependence than those of the complex (Figure 8). Furthermore, some *t*-STV-biotin complex junctions showed almost temperature-independent ETp with reversible behavior, which was not seen for *t*-STV junctions. Structural shrinkage at low temperatures can explain the negative temperature dependence (i.e., a relatively high current at low temperatures) of different STV junctions. At a low temperature, due to the proposed structural shrinkage, the effective thickness of the protein monolayer is expected to be reduced, which can explain the higher current through the junctions at lower temperatures. Upon biotin binding, the complex protein structure would be more conformationally rigid compared to biotin-free *t*-STV. Therefore, it can be assumed that biotin-free *t*-STV is structurally more flexible with more pronounced low temperature-induced structural shrinkage than the *t*-STV-biotin complex. The negative temperature dependence was observed for the junctions that passed a wide range of currents at a given voltage (Figure 7). As can be seen in Figures 7 and S9,S10, the extent to which currents were found to decrease with increasing temperature varied somewhat between junctions. This might be due to different *t*-STV orientations with respect to the substrate and the NW caused by the non-specific thiolation of the protein surface. In addition, some variation can originate from the above-mentioned possible variation in active (electrical) contact areas of the AuNW with the protein layer. Similar negative temperature dependence and its reversible behavior were also observed for *n*-STV and its complex (Figure S10).

The observed negative temperature dependence is similar to that of metals. Previously, strong, reversible negative temperature dependence was reported for an alkyl chain (non-protein) layer, where current has increased 30-fold (*I*_high_/*I*_low_) between 15 K (*I*_high_) and RT (*I*_low_).^38^ Based on IR and UPS data, this result was explained by decreasing the density of gauche defects, which can increase the layer’s molecular packing density with decreasing temperature, as well as freezing the molecular motion and decreasing vibration-electron coupling at a low temperature.^38,39^ A previous report on Cu(I)-Az in a Si-LOFO junction configuration showed that the applied positive bias caused a negative temperature dependence (*I*_80K_/*I*_300K_ ∼ 2) probably due to (reversible) protein structural deformation at a low temperature.^40^ The switch in temperature dependence of these Cu(I)-Az junctions with the polarity of the applied bias was explained by electrostatic effects.^40^ Recent work on Cu(II) Az junctions using Au with AuNW contacts^16^ detected very slight (*I*_80K_/*I*_300K_ ∼ 1.2) negative temperature dependence in the almost linear *I*–*V*–*T*, which, in light of the precision of the experimental measurements, was viewed as temperature independent. The present study shows a much stronger deviation from constant currents.

## 4 Conclusions

Results of ETp measurements are presented through junctions made with monolayers of two types of STV with and without biotin in a micro-electrode device configuration. While biotin binding was found to affect the protein conformation to a limited extent, no change in conductance via STV that is significant for the solid-state junction measurement configuration could be resolved as a result of ligand binding. This result implies that minimal changes in the protein frontier orbital energy levels occur with respect to the electrode Fermi levels. This is in contrast to the observed impact of binding electron-rich ligands, which was found to increase conductance by up to 2–3 orders of magnitude. Non-negligible, slightly metallic, negative temperature dependence is found for the majority of examined junctions with both types of STV and their biotin complexes, with an average two- to threefold increase in conductance upon lowering the temperature from 300 to 80 K for *t*-STV junctions. Such negative temperature dependence can be accounted for by a reversible temperature-induced structural shrinkage of the protein. Low temperature-induced, relatively high-conductive protein junctions (below 160 K) were correlated with the reduced solid-state immobilized protein layer’s thickness as an effect of structural shrinkage. Correcting for the effect of structural shrinkage leaves coherent tunneling transport as a possible ETp mechanism for the ∼3 nm thick STV monolayers.

## Supporting Information Available

Discussion of ellipsometric thickness measurement, AFM imaging, nano-shaving experiment with accurate contact force measurements, PM-IRRAS, CPD-derived work functions of the protein monolayers using Kelvin probe, and insights into the nanowire junction stability (PDF)
