# Supplementary material for: Biotin Binding Hardly Affects Electron Transport Efficiency across Streptavidin Solid-State Junctions
Source: Langmuir. 2023 Jan 17;39(4):1394–403. doi: 10.1021/acs.langmuir.2c02378 (PMC9893813; doi:10.1021/acs.langmuir.2c02378)
Supplement: Supplementary file 1 — la2c02378_si_001.pdf [file la2c02378_si_001.pdf]

Supporting Information

for

Biotin Binding Hardly Affects Electron Transport Efficiency across Streptavidin Solid-State Junctions

Sudipta Bera[a], Sharada Govinda[a], Jerry A. Fereiro[a,b], Israel Pecht[c]*, Mordechai Sheves[a]*, David Cahen[a]*

[a]*Department of Molecular Chemistry and Materials Science, Weizmann Institute of Science, Rehovot 7610001, Israel.*

[b]*The School of Chemistry, Indian Institute of Science Education and Research, Thiruvananthapuram, Maruthamala, Kerala 695551, India.*

[c]*Department of Immunology and Regenerative Biology, Weizmann Institute of Science, Rehovot 7610001, Israel.*

* *Corresponding Authors – email:* david.cahen@weizmann.ac.il; mudi.sheves@weizmann.ac.il; israel.pecht@weizmann.ac.il

1.1 Ellipsometry

Spectroscopic ellipsometry is a non-destructive, fast, indirect approach for assessing a thin film widths, based on a fitting model to the change in polarization amplitude (ψ) and phase (δ) of a signal reflected (in our case) from the thin film surface. This method requires a large sample area (1.2 × 0.5 cm) to accumulate the whole elliptical polarized incident light beam. In all our measurements, we maintain a fixed angle of incidence (70 °) over the wavelength range 350-1000 nm. The Cauchy model was used to get the average protein layer thickness by fitting of wavelength dependent ψ and δ plots using the in-built software in Woollam M-2000 V ellipsometry setup. First, a bare (solvent-cleaned, ozone-activated) Au substrate was measured, followed by a linker modified-Au, and with and without biotin modified protein on linker-coated Au for both *n*-STV and *t*-STV.

1.2 Atomic force microscopy

Atomic force microscopy (AFM) can serve to characterize ultra-thin films of soft materials such as proteins. Two back-to-back AFM imaging techniques have been employed, using a Bruker AFM setup (*Nanoscope V Multimode AFM*). First, AFM tapping mode imaging was performed with a moderately stiff Si_3N_4 AFM cantilever (spring constant 2.0-2.2 nN/nm) with resonance frequency 60-80 kHz. After identifying an aggregate-free, uniform protein

layer (by tapping mode topography), we switched to the contact mode for AFM nano-shaving, using the same AFM tip without changing the sample position. The spring constant of the AFM cantilever was calibrated by deflection sensitivity (α) calculation and thermal-K tuning operation. The exact contact force[1] applied by the AFM cantilever was calculated using the value of deflection sensitivity (reciprocal of the slope obtained from the linear part of the force-distance curve in the repulsive regime) and the thermal-K - derived spring constant (k) of the AFM cantilever:

$$\text{Contact force [nN]} = (\text{Setpoint} - \text{Free deflection [volt]}) \times \alpha\ [\text{nm/volt}] \times k[\text{nN/nm}]$$

Nano-shaving is a kind of (high force-induced) contact mode imaging, where the AFM-tip removes surface molecules (proteins in our case) from a much harder underlying substrate. For the *t*-STV monolayers, the nano-shaving was performed over ~ 500×500 nm-square scan area with 130-150 nN[1] applied contact force, which was effective for the removal of *t*-STV/*n*-STV proteins from the Au-substrate. However, we found that removal of *n*-STV molecules was relatively difficult, even with higher applied contact force. In order to get a clear topographic view of the nano-shaved area, we switched to the tapping mode after the nano-shaving of the protein layer. The thickness of protein film was obtained from the line-profile at the nano-shaved region of tapping mode AFM image (with 3× 3 μm-square scan area). *Gwyddion-2.58* software was used for processing the AFM images.

1.3 Polarization modulation-infrared reflection-absorption spectroscopy (PM-IRRAS)

For PM-IRRAS characterization of our protein monolayers, we used the PEM module of a single-channel Nicolet 6700 spectrometer with a grazing angle accessory and a liquid nitrogen cooled MCT detector. The collected IR spectrum for each sample was the average signal over 2000 scans at 0.8 cm^{-1} resolution with an incident angle of 80 degree. Dedicated *Omnic* 8.1 software served for PM-IRRAS data processing. In this work, we focused mainly on the amide-I and amide-II bands and their relative positions. We disentangled the influence of biotin binding on the STV spectrum by deconvoluting the bands, as explained in main text.

1.4 Surface potential measurements- Kelvin probe

Kelvin probe (KP)-based surface potential measurements allow for estimating the work function (ϕ) of a surface, relative to a surface with known (and ambient -stable) reference work function. Our main goal was to check how the different streptavidin surface charge affects the work function of the protein films. We used a macroscopic KP system from

Besocke (with gold gauze tip), operated inside a N_2-filled glovebox at room temperature. For every set of experiments, we first performed the measurement of the contact potential difference (V_{CPD} in volts) between the KP(Au tip) probe and freshly peeled highly oriented pyrolytic graphite (HOPG, the reference) surface. This was followed by the V_{CPD} (in volts) measurement between the KP tip and the surface of protein films. The relative work-function of the protein film was obtained from

$$\phi_{Sample}(eV) = \phi_{HOPG}[eV)] - V_{CPD}(HOPG) + V_{CPD}\ (Sample)$$

where the reference has a known value: $\phi_{HOPG} = 4.6\ eV$.[2] Here V_{CPD} (HOPG) and V_{CPD} (Sample) are the contact potential difference between HOPG surface and KP tip and the sample surface relative to KP tip, respectively.

1.5 Insights into Au-*protein*-AuNW junction stability

We found, that the junction (in)stability can often be assessed from the (ir)reversibility of temperature-dependent I-V measurements between the obtained results by cooling down versus heating up of the protein sample. Out of 40 *n*-STV junctions, only 20% enabled to measure the full temperature dependence (300K-80K); remaining junctions were either shorted or became open, i.e., lost contact, after a few I-V sweeps. The successful reversible temperature-dependent I-V responses were obtained between 300 and 110K are shown in Figure S10. We ascribed relatively higher (> 60%) yield of *t*-STV junctions compare to *n*-STV, towards the successful reversible temperature dependence study. That can be accounted by the poor monolayers quality of *n*-STV as discussed in main text.

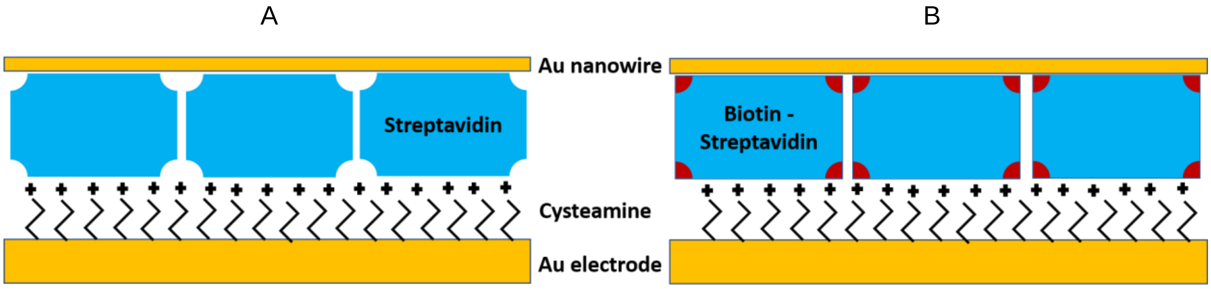

Figure S1: Scheme of nanowire aligned junction for (A) ***n*-STV**, and (B) ***n*-STV complex** (of biotin) in the configuration Au-Cys-(*n*-STV)-AuNW, where 'Cys' is cysteamine.

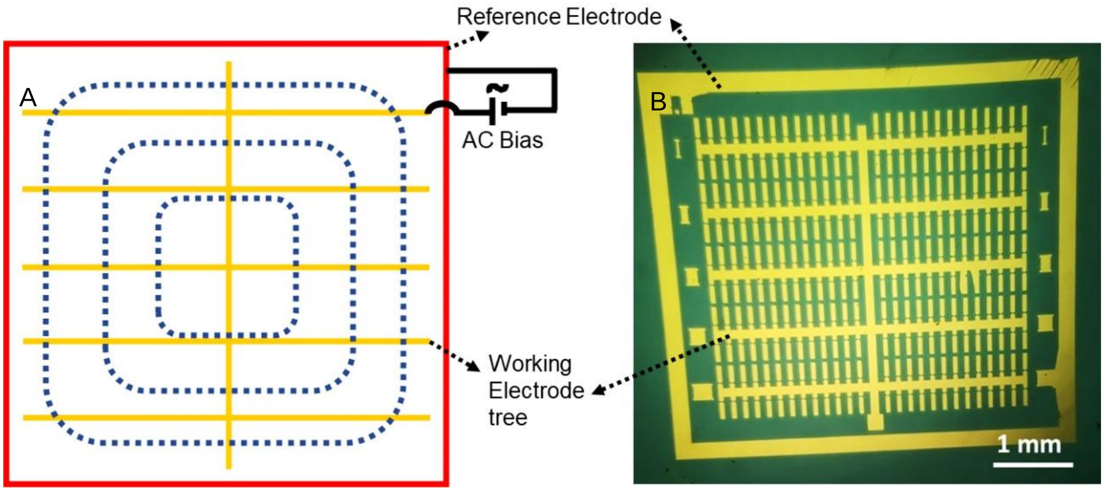

Figure S2: Microelectrode-chip diagram (**A**): AC bias is applied between the reference (red line) and the working (orange lines) electrodes; all working electrodes are connected together. Working electrodes cover a large area (~ 5 × 5 mm^2) that results in some variation of the AC field (applied AC volt/separating distance, relative to reference electrode) over the chip area. Each concentric dotted square (blue) represents a uniform AC field zone, where; the field varies for different dotted square. (**B**) photograph of a real microelectrode chip.

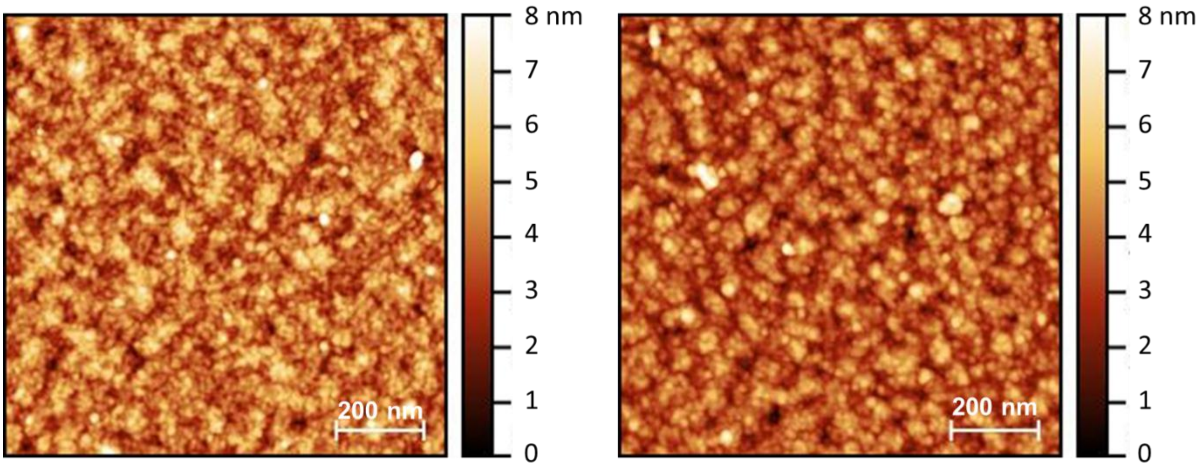

Figure S3: AFM topography of protein monolayers; (left) ***n*-STV** (*rms* roughness 0.83 nm) and (right) ***n*-STV complex** (*rms* roughness 0.8 nm) on gold (Au/Si substrate).

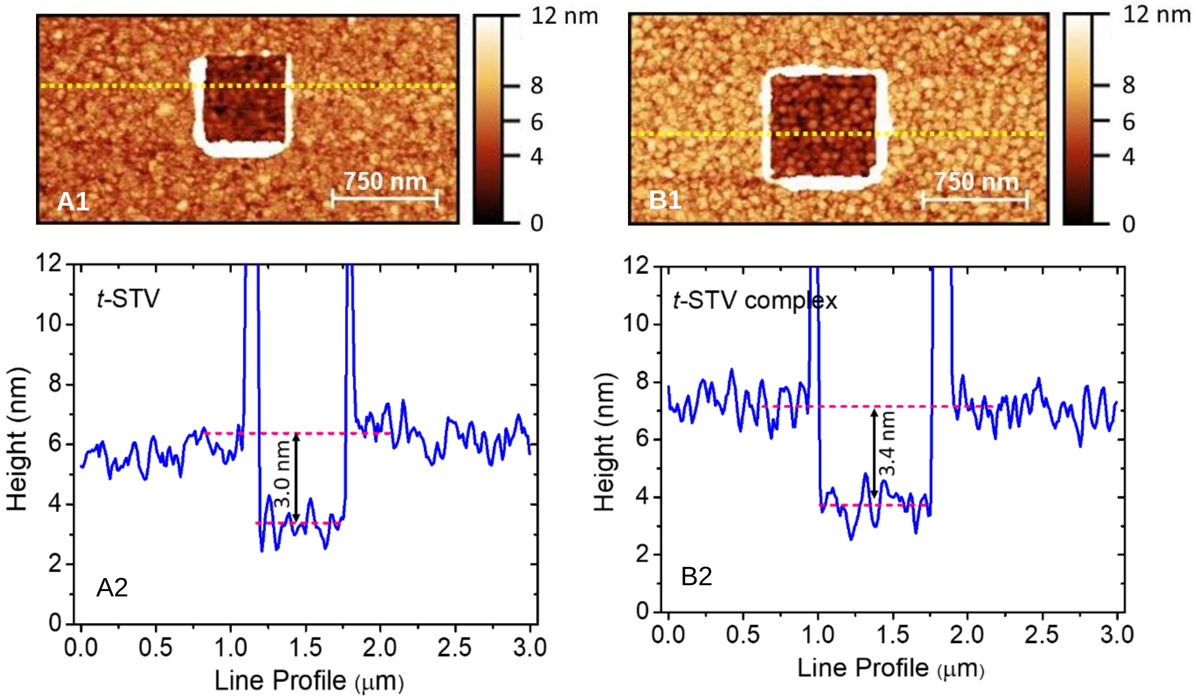

Figure S4: Example of nano-shaved AFM image of (A1) ***t*-STV** and (B1) ***t*-STV complex** (of biotin) monolayer on gold. The line profiles across the nano-shaved region are shown for (A2) *t*-STV and (B2) *t*-STV complex monolayer respectively.

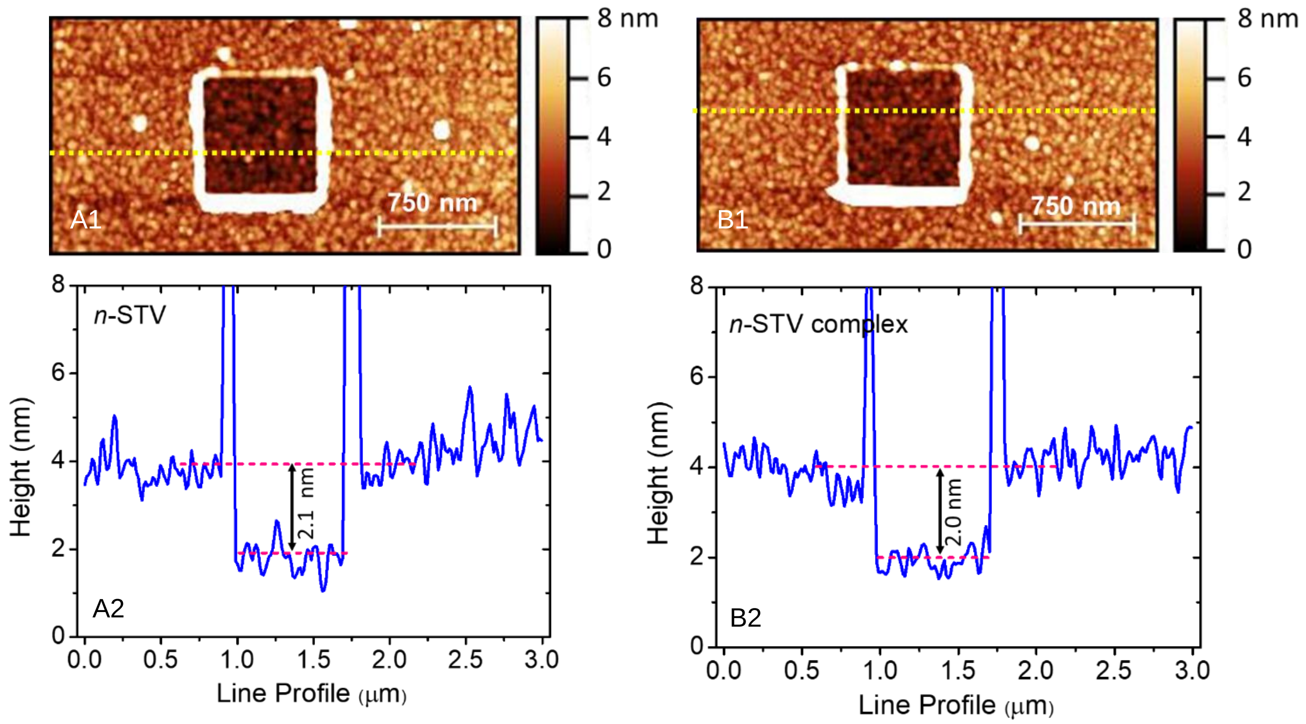

Figure S5: Example of nano-shaved AFM image of (A1) ***n*-STV** and (B1) the ***n*-STV complex** (of biotin) monolayers on gold. The line profiles across the nano-shaved regions are shown for (A2) *n*-STV and (B2) the *n*-STV complex monolayer respectively.

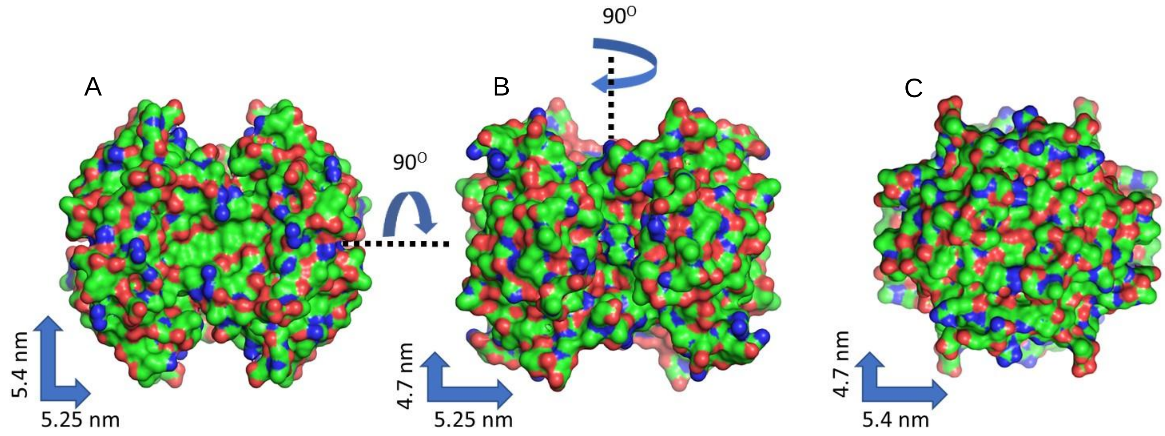

Figure S6: Different 2D views of streptavidin's 3-dimensonal structure (PDB 6J6K) with three perpendicular dimensions of 4.7, 5.25, and 5.4 nm (using PyMOL). Image 'B' was obtained by 90° rotation (along the horizontal axis, indicated by the black dotted line) of image 'A'. Similarly, image 'C' was obtained by 90° rotation (along the vertical axis indicated by the black dotted line) of image 'B'. In each image the vertical and horizontal protein dimensions are indicated.

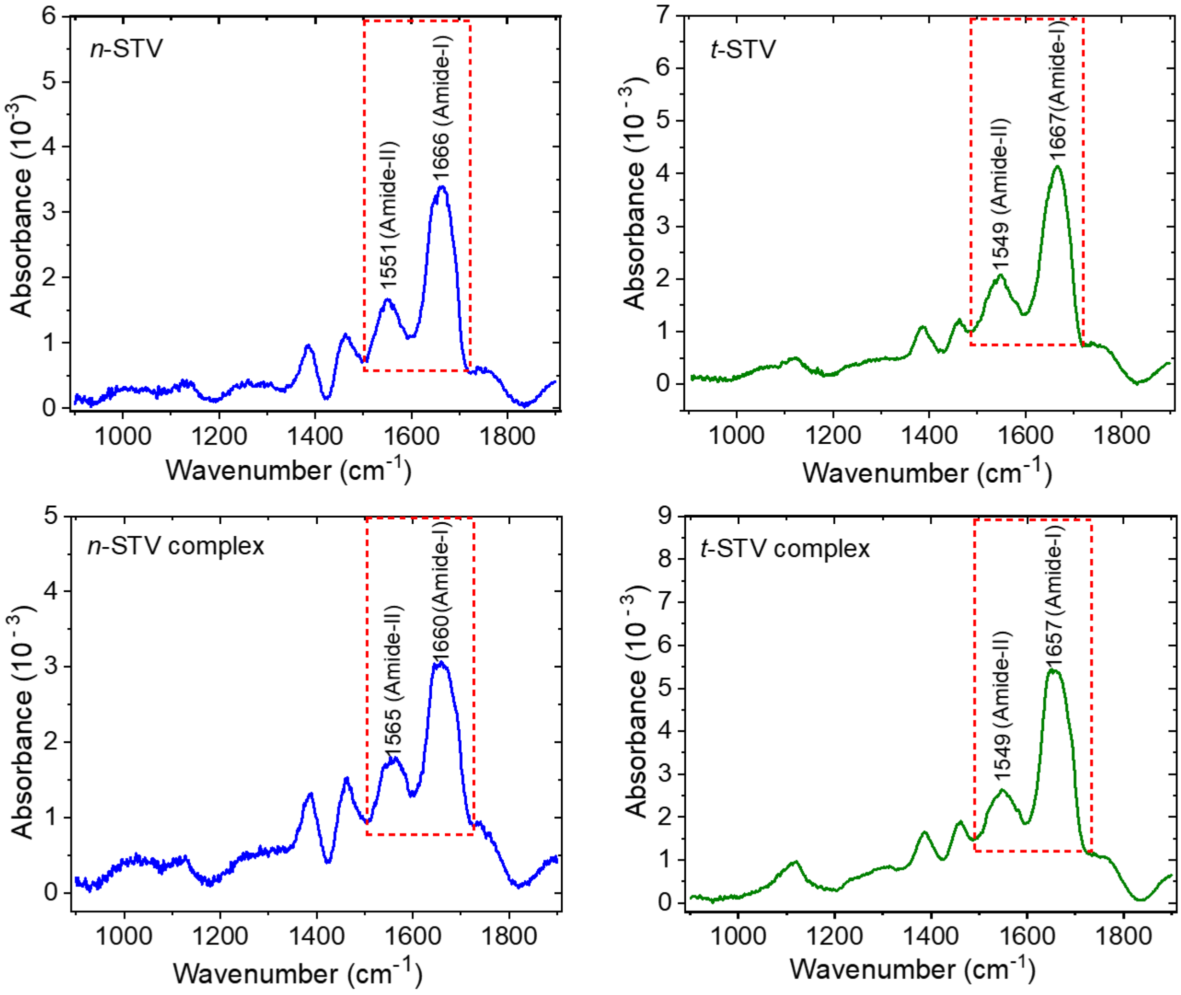

Figure S7: PM-IRRAS data of different protein monolayers (mentioned in each figure) on gold. An arbitrary unit is presented for absorbance. The amide-I and amide-II peaks (within dotted rectangular-box) have been highlighted for the comparison, which is explained in details in main text and figures.

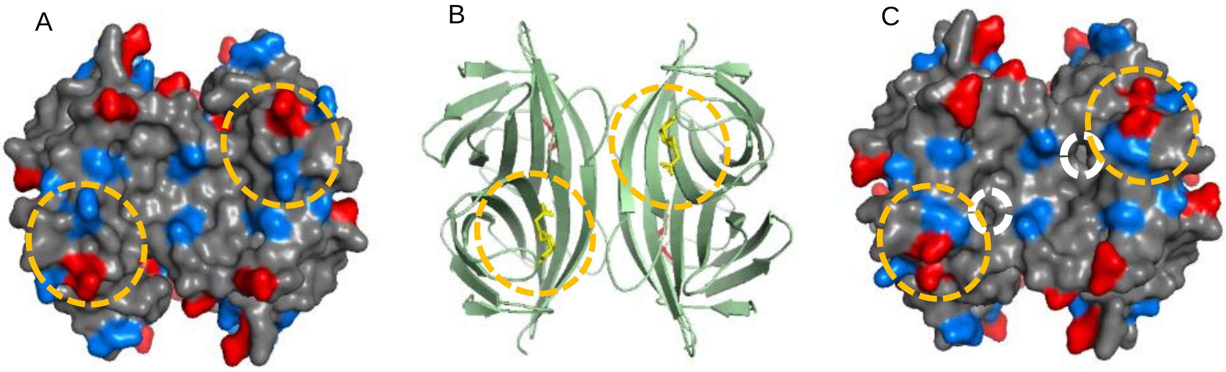

Figure S8: Surface charges (analyzed by PyMOL) of (A) *n*-STV (PDB-6J6K) and (B and C) its biotin complex (PDB-6J6J) with surface exposed acidic (red) and basic (blue) amino acid residues. Orange circles indicate the position of embedded biotins for two of *n*-STV subunits (from the top view, as shown) and the white circle represents the surface exposed biotin binding pocket of *n*-STV after biotin binding.

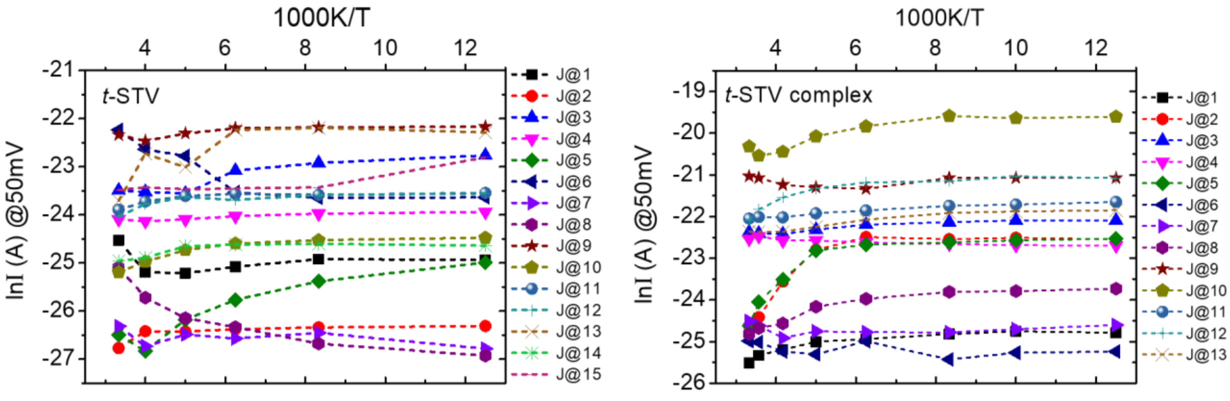

Figure S9: ln(I) – (1/T) characteristics of Au-(*t*-STV)-AuNW junctions for the covalently bound ***t*-STV** (left) and ***t*-STV complex** (of biotin) (right) monolayers on gold over the temperature range 300K to 80K (@ 50 mV applied bias).

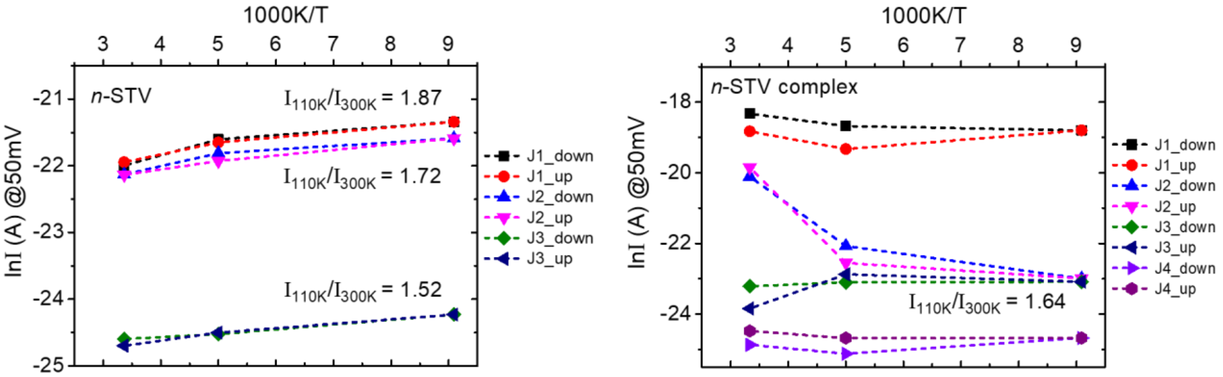

Figure S10: Reversible ln(I) – (1/T) characteristics of ***n*-STV** (left) and ***n*-STV complex** (of biotin) **(**right) junctions over the temperature range 300K to 110K under 50 mV applied bias. Here ‘down’ refers to cooling down to 110 K and ‘up’ refers to heating up to room temperature.

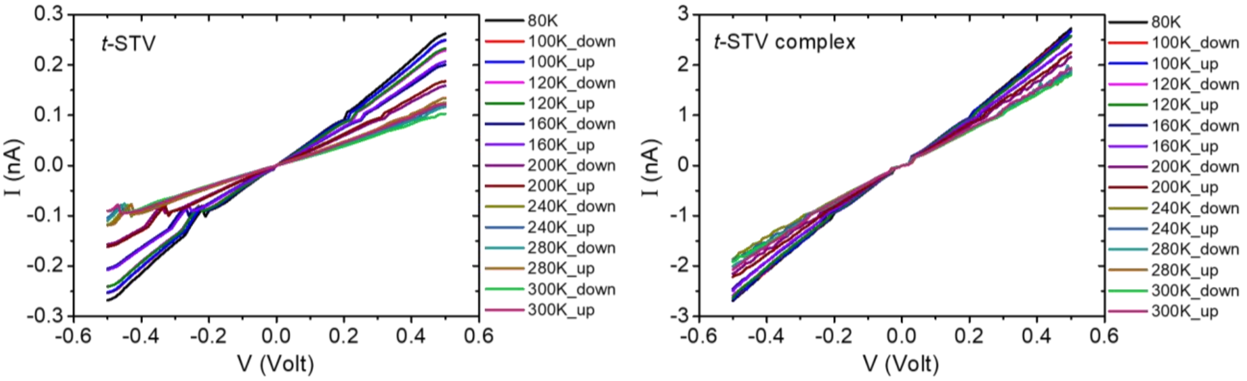

Figure S11: Reversible current-voltage response (as a function of temperature) of the representative junctions (over the bias sweep ± 0.5 V) of ***t*-STV** and the ***t*-STV complex** (of biotin) monolayers; ‘down’ means cooling down to 80 K and ‘up’ means heating up to room temperature.

References

(1) Bera, S.; Kolay, J.; Banerjee, S.; Mukhopadhyay, R. Nanoscale On-Silico Electron Transport via Ferritins. *Langmuir* **2017**, *33*, 1951–1958.

(2) Fernández Garrillo, P. A.; Grévin, B.; Chevalier, N.; Borowik, Ł. Calibrated Work Function Mapping by Kelvin Probe Force Microscopy. *Rev. Sci. Instrum.* **2018**, *89*, 043702.
